# Systems Science in Nutrition and Dietetics Research: A Practical Lens for Applying Systems Approaches in Research

**DOI:** 10.1111/jhn.70281

**Published:** 2026-06-11

**Authors:** Kiara Too, Adam Hulme, Amy Kirkegaard, Mark Robinson, Lauren Ball

**Affiliations:** ^1^ Centre for Community Health and Wellbeing The University of Queensland Brisbane Queensland Australia; ^2^ Southern Queensland Rural Health The University of Queensland Brisbane Queensland Australia; ^3^ Institute of Social Science Research The University of Queensland Brisbane Queensland Australia

**Keywords:** complexity, nutrition and dietetics, research, systems science

## Abstract

Research paradigms in nutrition and dietetics research have remained largely hegemonic and reductionistic, leading to interventions that inadequately address complex real‐world challenges, such as diet‐related chronic diseases. These paradigms have placed emphasis on individual‐level interventions that insufficiently account for the evolving individual, environmental, sociocultural and political contexts that shape dietary behaviour and related health outcomes. Viewing topics and problems through a complex systems lens reveals that health and behaviours are influenced by multiple, context‐dependent factors that interact and evolve over time. Achieving meaningful improvements in health outcomes therefore requires cross‐disciplinary collaboration to build a more complete understanding of a problem and identify the factors and interrelationships driving systemic behaviour over time. The continuing emergence and application of systems science methods highlight the value in reconsidering the application of a complex systems approach to complement traditional research paradigms within nutrition and dietetics research. This paper provides an overview of the opportunities that systems science offers the nutrition and dietetics community and describes how its principles and methods have been used in research, including example areas for future application. Three commonly utilised system science approaches are explored: agent‐based modelling, system dynamics, and network analysis. While translating system insights into practice is necessary for achieving real‐world change, this paper focuses on practical entry points for getting started in keeping with the aims of this special issue on advancing systems‐based practice in nutrition and dietetics. By continuing to embrace systems thinking, the nutrition and dietetics research workforce can strengthen its capacity to understand complexity, design coordinated actions and drive transformative, sustainable improvements in population nutrition‐related health.

## Summary

- This article encourages the need for greater engagement with and confidence in using systems science approaches to understand and promote change for complex nutrition and dietetic problems.
- Agent‐based modelling, system dynamics, and network analysis are three approaches that can explore the impact of micro‐level behaviour, social dynamics and system structure within the nutrition and dietetics field.
- We propose three guiding principles to enhance early engagement with system science methods, including methods awareness, alignment with the problem space, and integration into education.

## 1 Introduction

The inherent complexity of modern life challenges researchers and clinicians worldwide. This complexity also applies to nutrition and dietetics professionals who work in areas such as nutritional science, clinical nutrition, food services, sports nutrition, primary care, public health, and research and academia. These professionals face issues shaped by many interrelated factors that exist within broader environments and are influenced by forces operating at various levels. Thus, meaningful progress in nutrition and dietetics broadly necessitates collaboration across systems and disciplines to address the underlying factors that collectively shape dietary health outcomes.

Complexity science principles highlight that food and population health systems, and diet‐related behaviours are shaped by multiple, context‐dependent, interacting factors that together form dynamic systems which adapt and change over time [1, 2, 3]. Understanding the interplay within and across individual, environmental, sociocultural, and political systems emphasises that problems that persist over time cannot be addressed sustainably by any single discipline or sector alone. Rather, both problems and solutions are best viewed as interconnected systems of people, processes, ideas and physical structures [4]. Facilitating system‐wide change calls for methodological approaches that cut across disciplines and engage communities and various stakeholders to make sense of real‐world complexity. Improving the ability to navigate seemingly intractable and longstanding nutrition and dietetic challenges as a workforce will advance nutrition‐related health and wellbeing and contribute meaningfully to population outcomes.

To address challenges arising from this complexity, research must remain one step ahead by complementing traditional scientific approaches focussed on individual factors with systems‐orientated paradigms that capture the dynamic interrelationships driving nutrition and health outcomes [2]. Systems science refers to an interdisciplinary field that uses a range of theories, concepts, and methods to conceptualise, explore and analyse complex systems [5]. Promisingly, there is a growing trend in the application of systems science in nutrition and dietetics, with 2019 marking a turning point in scientific attention. This is reflected in PubMed trends, where articles referring to (“systems science” or “systems thinking”) [Ti/Ab] and (“nutrition” or “diet*” or “food”) [Ti/Ab] have grown from ~75 in 2019 to > 200 in 2025, an almost threefold increase. Systems‐focused research in nutrition and dietetics has examined factors influencing children's dietary choices [6, 7], diet in low‐income groups [8], diet‐related health outcomes [9, 10], ultra‐processed food consumption [11], food environments [12, 13], and trends in topical research areas [14], as well as how networks shape individual dietary behaviour [15], prevention efforts [16], program implementation [17] and health system networks [18]. In addition, systems modelling has enabled researchers to assess the implications of interventions and policies for sweetened beverage consumption [19, 20], food accessibility [21], and fruit and vegetable intake [22] over time.

Despite rising trends of systems science in nutrition and dietetics, continued over‐reliance on traditional scientific approaches could be limiting the discipline's capacity to understand and address complex challenges. The value of systems‐based approaches has been recognised within public health nutrition [23, 24] and food systems [25], but there remains a need to encourage greater exposure and practical utilisation of these theories, concepts and methods for complex problems. Greater engagement with and integration of established approaches within research are required to build on existing exemplars and strengthen the capacity for complex problem solving [2, 26].

The aim of this paper is to contribute to the ongoing narrative about the opportunities that systems science offers the nutrition and dietetics community and to outline how three approaches have been applied in research, with examples areas for future applications. Specifically, this contribution is tailored for nutrition and dietetics researchers interested in gaining awareness of, or using, systems science in their work, and provides a brief introduction, suggested protocols (including key resources), and practical tips for getting started. By embracing systems science, the profession can strengthen its capacity to understand complexity, guide targeted and impactful action, and drive transformative, sustainable improvements in population nutrition and health.

### 1.1 Defining a Complex System

Determining the opportunities and benefits that systems‐based approaches offer for nutrition and dietetics requires us to first consider the relevant contexts and philosophical underpinnings. Complexity cannot necessarily be conveyed by a simple definition but instead is represented by broad characteristics (Table 1). Characteristics identified through tools like the Cynefin Framework [3] assist in distinguishing between simplicity and complexity, marking a valuable initial step in the process of inquiry. For instance, providing nutrition advice for iron‐deficiency anaemia can range from a simple recommendation, such as increasing daily iron intake to restore stores, to addressing significantly more complex factors like timing and distribution, bioavailability of heme versus non‐heme iron, nutrient interactions, and individual preferences.

**Table 1:** Characteristics of complex systems [1, 27].

Characteristic	Description	Examples in nutrition & dietetics
Interactions between diverse components	Many heterogenous elements that demonstrate complex interactions, including non‐linearity (i.e., small changes having large effects and vice versa) and feedback loops, where an element feeds back into itself (feedback can be reinforcing i.e., amplify the behaviour, or balancing i.e., counteract the behaviour).	The human body: Consists of multiple different organs (e.g., liver, stomach, pancreas) that interact to digest food and regulate metabolism. These interactions are non‐linear, e.g., a small amount of food can trigger a large response in catabolic hormones, which rely on feedback loops to maintain energy stores. Dietary behaviour: Influenced by the interaction of diverse factors, e.g., nutrient quality, meal timing, psychological aspects (such as emotional eating), and socioeconomic status. Non‐linear interactions could include significant changes in mood having small effects on diet quality, with feedback loops indicating how changes in energy levels reinforce the impacts of poor eating patterns to promote change. Healthcare organisations: Include various interacting elements, e.g., health practitioners, administration staff, patients and policy frameworks to deliver nutrition care. Non‐linearity and feedback describes how small policy change can lead to large shifts in patient outcomes and workforce response to the feedback (such as increased patient readmissions) to maintain service quality.
Emergent properties	Broader system pattens/behaviour that cannot be predicted from the individual elements.	Glycaemic control: Emerges from the combined influence of medication adherence, type and distribution of carbohydrate intake, stress management and/or physical activity. None of the elements individually determine stable blood glucose control. Gut microbiome: Result from interactions among fibre intake, pre‐ and probiotics, stress, physical activity, sleep, and antibiotics use. Overall microbial diversity and resilience cannot be predicted from any single factor. Hospital patient satisfaction and nutrient adequacy: Emerges from menu planning, cultural food preferences, food presentation, and staff training. Improving one factor alone does not ensure high satisfaction and nutrition adequacy.
Adaptation and self‐organisation	The effect persists over time, and complex systems self‐organise and adapt to changing circumstances.	Bone health: Adequate calcium and vitamin D intake supports bone density over years, but hormonal changes or reduced intake leads to the body adapting by increasing bone resorption to maintain blood calcium levels. Dietary behaviours: Individuals employ strategies to improve their dietary behaviours, and when exposed to challenges (e.g., increased stress, reduced time) adapt their behaviours given the new constraints (e.g., quick meal options) to maintain diet quality. Interdisciplinary collaboration: Working closely with other health practitioners in an organisation enhances patient outcomes, but if the team structure changes or priorities shift dietitians will use their own resources and processes to maintain patient outcomes.
Open boundaries	Open systems that interact with their environment. Complex systems have no boundary, but we impose one for the purpose of exploring the system.	Clinical dietetic practice: Evaluating patient outcomes in a primary/tertiary setting requires a boundary around the organisation of interest; however, the system will interact with external influences, such as new guidelines, altered funding schemes and workforce changes. Public health nutrition programs: Community programs focus on improving nutrition outcomes, but interact with policy shifts, economic conditions and population health trends that influence these focuses. Individual dietary behaviour: Individuals focus on personal eating habits, including optimising diet quality within time and financial constants and personal preferences, but this system is also shaped by food availability, social networks, cultural norms, and marketing.
Dynamic equilibria	Complex systems operate far from equilibrium, continually shifting towards and away from acceptable boundaries.	Human metabolism: The body is constantly shifting between anabolic and catabolic process, rather than staying in a fixed metabolic state. Healthcare systems: Constantly adapting to internal and external changes, including policy and patient needs. Sports nutrition: An athlete's diet is continually changing in response to training load, competition schedule and climate.

*Note:* The characteristics listed are illustrative of selected key tenets of complexity and are not intended to represent an exhaustive account of the field.

The distinction of complex systems acknowledges that the interactions between system components prove challenging for generating system‐wide change. Systems‐based approaches emerged from a recognised need to step back and “look at the bigger picture”, to otherwise acknowledge the whole is more than the sum of its parts [5]. The field recognises that understanding complex problems requires researchers to leverage such approaches to strengthen their capacity for positive impact [28].

### 1.2 Philosophical Approach

While systems science provides practical tools, “systems thinking” refers to the underlying worldview and philosophical approach that shapes how humans understand complexity. Systems thinking developed from ongoing tension between mechanism and holism, stimulated by Thomas Kuhn's revolutionary research philosophy in 1962 [29]. This prompted the formalisation of systems thinking by Richmond, describing it as an art and science allowing us to see the ‘trees’ and the ‘forest’ simultaneously [30]. While systems thinking is closely aligned with holism, it does not reject reductionism but critiques the overreliance on reductionistic approaches that tend to overlook complexity under specific conditions. Thus, systems thinking provides a duality of the narrow and broad paths to health, providing opportunities to optimise individual health and address equity and population wellbeing [31]. Box 1 briefly describes the origins of systems science.

Box 1 **Origins of systems science.**

The history of systems science is diverse and complex, spanning multiple disciplines [32]. It is widely acknowledged that the field emerged from biology, with key pioneers developing frameworks depicting the integrated wholes of living organisms and explaining universal principles governing both natural and social systems. These include Bogdanov's Tektology and Bertalanffy's General System Theory [33]. Another major contribution included Wiener's Cybernetics, which studies control and communication in animals and machines and introduced the role of feedback in self‐regulating systems [34]. During the 1950s and 60s, systems thinking had a strong influence on engineering and management as a tool to solve practical problems but has been increasingly integrated across multiple disciplines, including health [35].

In nutrition and dietetics, systems thinking offers a complementary perspective by examining individual‐level dietary intake and diet quality alongside the broader environmental, economic, sociocultural and political factors that shape food choices and nutrition outcomes. Reductionism would aim to simplify dietary behaviour to nutritional composition, e.g., food group analysis, macronutrient distribution or micronutrient diversity. Whereas holism acknowledges that dietary behaviour is more than the sum of those parts, considering the individual behavioural patterns, economic (e.g., food prices), social (e.g., school or workplace norms), cultural (e.g., cultural food preferences and practices) and political (e.g., regulation or food labelling laws) factors which interact and evolve over time.

## 2 Relevant Systems Science Approaches for Nutrition and Dietetics

More broadly, public health has experienced rapid growth in the breadth of systems science approaches employed, with common approaches including agent‐based modelling (ABM), system dynamics and network analysis [27, 32]. These approaches are also relevant for making sense of complexity in nutrition and dietetics, as they capture different dimensions of complexity, including the impact of micro‐level interactions on macro‐level behaviour (i.e., agent‐based modelling), social dynamics at the meso‐level (i.e., system dynamics), and the typology and structural connectivity of systems (i.e., network analysis).

The nutrition and dietetics workforce seeks to understand how to effectively and efficiently improve nutrition outcomes for individuals, communities, and society. Complexity exists across all areas of nutrition and dietetics, and the most appropriate approach depends on the type of problem and anticipated outcomes. Table 2 outlines the three system science approaches described, including key resources, a suggested protocol, and nutrition and dietetic specific example areas for future application. These key points have been synthesised from recognised protocols in the broader literature and is not intended to be a complete representation of the respective approaches. The table aims to provide an entry point build awareness and consider how to engage with the methods.

**Table 2:** Overview of relevant systems science methods and the application to nutrition and dietetics.

Approach	Overview and key resources	Suggested protocol	Applications to nutrition and dietetics
Agent‐based modelling	Agent‐based modelling explores how individual agents' interactions and rules generate emergent system‐level behaviours, making it valuable for predicting intervention impacts and population health dynamics. Key resources: *An Introduction to Agent‐Based Modeling: Modeling Natural, Social, and Engineered Complex Systems with NetLogo* [36] *Agent‐Based and Individual‐Based Modeling: A Practical Introduction* [37] *Agent‐Based Models* [38]	Proposed protocol [39] 1. *Problem articulation* Define the research question and determine if agent‐based modelling is appropriate Define the behaviour being explored and the system it emerges from 2. *Design the model* Identify the scope of the model, i.e. what aspects of the complex system will be described Define the classes of and quantity of agents included (e.g., people, organisations) and the properties that describe the agent Define the agents' behaviours and environment Define the input and outputs of the model Determine the time step, which includes initialisation (i.e., creating the agents and network) and an iteration time (i.e., time when each agent determines if it will adopt the output behaviour). The model terminates when all agents have adopted the behaviour 3. *Construct the model* Develop computational model and code into agent‐based modelling platform (such as NetLogo, Repast, Mason, or Swarm) Initialise the model by creating the agents, set them to the default state (i.e., behaviour not adopted) and connect the network. Adoption decision refers to agents deciding to adopt the behaviour Collect statistics on number of agents who have adopted the behaviour Repeat if agents are yet to adopt the behaviour, otherwise terminate 4. *Analyse the model* Repeat simulation under different input and output parameters and analyse the results to verify and validate the model 5. *Model interpretation* Evaluate model outcomes: Were the outcomes expected/unexpected? Do they reflect patterns observed in the real world? How did the model respond to change?	Existing examples in nutrition and dietetics: Model the potential impact of interventions that reduce sugar‐sweetened beverage availability [20] Evaluate the effect of interactions between household and environmental factors on food security, and test impact of interventions focusing on food availability [21] Assessing the potential impact of interventions aiming to improve access and reduce price of fruit and vegetable across different neighbourhoods [22] Potential future research areas: How do dietitians adapt their practice when healthcare policies or resource allocations change? What mechanisms at the individual‐interaction level drive the emergence of chronic disease in a community? How do universities with different resource levels respond to changes in accreditation requirements for nutrition and dietetic programs?
System dynamics	System dynamics models and explores feedback loops, stocks and flows, and time delays, enabling researchers to test scenarios, identify potential leverage points and policy options, and explore system‐wide impacts of interventions over time. Key resources include: *Industrial Dynamics* [40] *Thinking in Systems: A Primer* [5] *Business Dynamics: Systems Thinking and Modelling for a Complex World* [41] *Modelling the Environment: An Introduction to System Dynamics Modelling of Environmental Systems* [42] *Strategic Modelling and Business Dynamics; A Feedback Systems Approach* [43]	Steps of the modelling process [41] 1. *Problem articulation* What is the problem, and why is it a problem? What are the key variables and concepts we must consider? How far back in the past lie the roots of the problem? How far into the future should we consider? What is the historical behaviour of the key concepts and variables? What might their behaviour be in the future? 2. *Formulation of a dynamic hypothesis* What are the current theories of the problematic behaviour? Formulate a dynamic hypothesis that explains the feedback structure underpinning system behaviour Map causal structure, e.g., stock and flow map, causal loop diagram, and subsystem diagrams 3. *Formulation of a simulation model* Specify structure and decision rules Estimate parameters, behavioural relationships and initial conditions Test for consistency with the purpose and boundary Software options include Vensim, AnyLogic and STELLA Architect 4. *Testing* Determine if the model produces the system behaviour observed Does the model behave realistically when stressed by extreme conditions? How does the model behave, given uncertainty in parameters, initial conditions, model boundary and aggregation? 5. *Policy design and evaluation* Facilitate intervention/policy design to see how change might be represented in the model Test “what if” scenarios to see how the model responds to system changes Test the interaction of interventions/policies to see how they interact	Existing examples that formulate a dynamic hypothesis: Community‐based workshops to explore children's nutrition and health [6] Examining the economic, social, physical and political food environments for low‐income groups through a literature review [8] Identifying the key actors, structures, incentives and dynamics underlying the global ultra‐processed food system [11] Examples that identify leverage points: Collaborating with stakeholders to understand the factors underlying a local food environment and identify leverage points [12, 13] Examples that formulate a simulation model: Engage community stakeholders to inform a model to increase water consumption and decrease sugar sweetened beverage in a regional town [19] Potential future research areas: What are the system factors and feedback loops influencing the transition of patients between community and hospital settings? How does the interaction between patient preferences, food service policies, and resource allocation affect overall food waste in hospital settings? How do changes in policy, training, and resource allocation influence the long‐term effectiveness of malnutrition prevention strategies in aged care?
Network analysis	Network analysis maps the structure and relationships among actors in a system to reveal patterns that influence behaviours and health‐related outcomes, helping identify opportunities for improved communication and coordination. Key resources: *Introduction to Graph Theory* [44] *Networks* [45] *Network Science* [46] *Social Network Analysis: Methods and Applications* [47] *Social Networks and Health: Models, Methods and Applications* [48]	Proposed protocol [45, 46] 1. *Problem articulation* Define the nodes (e.g., dietitians or hospitals) and edges, including whether they will show direction and/or weight (e.g., referrals) Define the scope, i.e., information that is included/excluded, (e.g., hospitals in a region) 2. *Collect relevant data based on the research question* Potential data sources include: ◦ Surveys/interviews asking people who they interact with (i.e., egocentric) or snowball sampling to follow connections ◦ Direct observation (e.g., observe interactions in hospitals) ◦ Data from archival or third‐party sources (e.g., academic literature, social media, emails, administrative records) ◦ Affiliations (e.g., membership in professional associations) 3. *Construct the model* Represent nodes and edges visually Software options include Graphviz, Social Network Visualizer, Pajek, Gephi, NetworkX, and yEd 4. *Analyse the network to understand which actors are most influential and how information flows. Common techniques including:* Overall network structure or shape, including random (i.e., connections spread equally); ‘small world’ (i.e., short edges between nodes); or scale‐free (i.e., few nodes have many connections) Centrality describes the most important/central nodes through considering the: ◦ Degree (i.e., how many connections a node has) ◦ Closeness (i.e., relationship to all other nodes in a network) ◦ Betweenness (i.e., how important a node is in the average pathway between other pairs of nodes), and ◦ Clustering (i.e., groups of nodes that are tightly connected)	Existing examples in nutrition and dietetics: Use a bibliometric analysis to explore trends in research topics, such as food security [14] Investigate the relationship between diet and depression in college students [10] Exploring how social networks shape dietary behaviours [15] Understanding prevention networks in a local government area [16] To depict, diagnose and evaluate networks in health systems [18] Potential future research questions: How do patterns of connectivity among food system actors influence resilience and efficiency in delivering nutritious foods? What clusters or communities exist within dietetics research networks, and how do they relate to knowledge dissemination? How do networks of dietitians use mutual recognition pathways to shape opportunities for international practice?

*Note:* This table is not intended to provide a comprehensive or exhaustive representation of the literature but rather compile proposed protocols and nutrition and dietetics relevant example areas for future application of the discussed systems‐based approaches.

### 2.1 Agent‐Based Modelling

ABM represents a “ground up” approach that uses computer simulation to illustrate how agents' (e.g., individuals, households, dietitians, private practice organisations, hospital and food services, or policymakers) give rise to broader system behaviour and emergent patterns [38]. In ABM, agents are given a set of rules *a priori*, which in the context of nutrition and dietetics may reflect taste preferences, financial considerations, clinical guidelines or organisational policies. The computational model allows researchers to simulate how interactions between agents and the environment influence individual decisions, leading to system‐wide adaptation and self‐organisation (e.g., changes in population dietary habits or hospital service outcomes) [38, 49]. Originally used to model infectious disease transmission, ABM has diversified to explore modelling the potential effects of interventions and has diverse applications for understanding how individual behaviours shape broader population health over time and predicting the potential large‐scale impact of interventions.

### 2.2 System Dynamics

System dynamics [40, 41] captures how the structure of a system shapes its behaviour over time from an endogenous perspective, while also recognising that simplified models and explicit assumptions are necessary to handle complexity and generate useful insights. System dynamics employs a “top‐down” approach, focusing on modelling the following elements:

- Stocks and flows: Stocks refer to quantities that accumulate over time (e.g., number of practising dietitians, patients awaiting care), and flows represent the rate of change per unit of time (e.g., annual increase in dietitian numbers, how quickly patients enter the treatment pool).
- Feedback loops: A closed chain of causal connections where the output of a system or system elements feed back into itself, which can reinforce behaviour (e.g., public health campaigns reinforcing populations positive attitudes towards nutritious foods), or balance behaviour (e.g., nutrition labelling reducing ultra‐processed food purchasing).
- Time delays: Represent that change takes time due to physical and material (e.g., hiring dietitians) or informational processes (e.g., spreading new clinical guidelines through professional networks).
- Variables: Endogenous variables are generated by the internal structure of the system, whereas exogenous variables are specified outside the model. These variables may be represented using constants, parameters, or graphical functions, including nonlinear relationships where effects change disproportionately across different values [41].

System dynamics modelling helps researchers explore how changes in one part of a system influence outcomes in other parts, identify potential leverage points for intervention, and track how behaviours may evolve over time [41]. For instance, expanding the number of dietitians at a primary practice may affect other areas of the system, such as increased client capacity, reduced wait time and improved nutrition‐outcomes. Although, this could also lead to unanticipated consequences [50], such as impaired quality of care due to inadequate professional development, increased operating costs, or employer dissatisfaction from reduced individual client load. System dynamics models can also help reveal likely points of leverage, which may show that frequent professional development and increased marketing to attract clients could improve revenue and quality of care. Applying system dynamics enables researchers to capture dynamic interactions that shape health outcomes and predict how changes in one area of the system influence system‐wide behaviour over time.

### 2.3 Network Analysis

Where agent‐based modelling explores the emergence of macro‐level patterns over time, network analysis maps, measures and analyses the relational structure of a system. Network analysis is grounded in graph theory, focusing on interactions between parts of a system and can be applied to any type of network, including technological, information, social, and biological [45]. In this method, a “node” represents an individual entity in a system, while edges (i.e., lines between nodes) depict connections between entities, which can show the direction or weight if necessary. In nutrition and dietetics, nodes could include people (e.g., dietitians or patients), organisations (e.g., hospitals or food retail outlets), information sources (e.g., clinical guidelines or educational resources), or programs/initiatives (e.g., public health campaigns or school meal programs). Edges describe the connection between nodes and could represent peer/professional relationships, information exchange, economic transactions, or communication patterns. Mapping networks allows researchers to analyse network properties, such as structure/shape and centrality (i.e., the importance of a node based on closeness and connections to other nodes) [45]. In nutrition and dietetics, this could reveal how information disseminated through professional and social networks influence dietary habits, or how connections between factors relate to the increased risk of non‐communicable diseases. By highlighting the structure and strength of these connections, network analysis helps identify opportunities to improve communication, collaboration, and coordination within nutrition and dietetic systems. These insights are valuable for designing interventions that leverage social influence, strengthen professional networks and optimise health system performance to improve nutrition outcomes.

## 3 Getting Started in Systems Science

**Figure 1:**
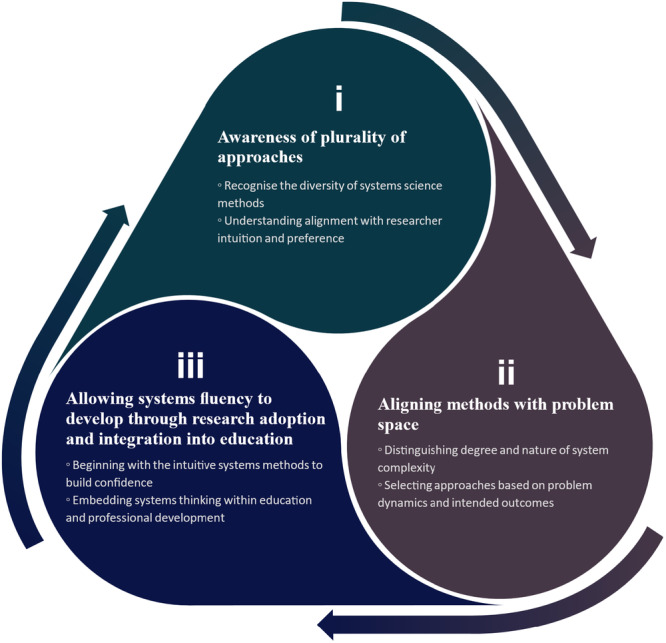
Authors' three guiding principles for getting started in using systems science presented as a cyclical process, highlighting how each principles facilitates the following, with education and professional development further stimulating greater awareness of approaches to strengthen future systems‐informed research.

For researchers, educators and practitioners alike, getting started in systems science may feel somewhat overwhelming, particularly when confronting issues of complexity, multiple interactions and dynamic change. Although there is no single, straightforward pathway into systems science, we propose the following three guiding principles (as shown in Figure 1) that will enhance early engagement and build confidence in nutrition and dietetics research: (i) awareness of the plurality of approaches; (ii) aligning methods with the problem space; and (iii) allowing systems fluency to develop through research adoption and integration into education. George Box's quote that “all models are wrong, some models are useful”, highlights that models are not intended to accurately replicate the real world, but rather to provide a tool for articulating, understanding, and discussing complex problems. Therefore, a “useful model” is critiqued based on the practical utility in assisting to address the complex problem, rather than the accuracy [51].

First, we advocate researchers start by developing a broad awareness of the methodological landscape to appreciate the diversity of available systems approaches, without necessarily having to ‘master’ all at once. Systems science encompasses a range of methods, including and extending beyond the three discussed in this paper that support mental model elicitation, system conceptualisation and qualitative mapping through to discrete event modelling and quantitative simulation. Therefore, value is found in referring the reader to additional resources that signify the practical implications of systems thinking and provide comprehensive guidance on a variety of approaches [52] or describe specific subsets of approaches, such as causal mapping [53] and soft systems techniques [51]. Additional works describe the relevance of systems thinking approaches for real‐world contexts, including stimulating social change [54], complex global crises [55], and making decisions in the face of complexity [56].

Furthermore, because no single method is inherently “superior”, researchers may benefit from recognising which systems science theories and methodological styles resonate most with their own intuition, analytical preferences and preferred ways of thinking. Being aware of this alignment will make the early phases of engagement with systems science more manageable and meaningful and contribute to building a personal orientation toward the field. This is particularly relevant to nutrition and dietetics researchers, as the field faces a variety of complex problems that span individuals, communities, and broader populations. Therefore, the field will benefit from a diverse skillset within researchers across a variety of systems approaches to capture and address the breadth of challenges we face, e.g., exploring nutrition‐related behaviours, collaborating across disciplines to improve the quality of health care, mapping food environments, or assessing the impact of public health initiatives.

A second consideration pertains to the problem space itself. Indeed, distinguishing the degree and type of complexity of the “problem” of interest (e.g., childhood dietary choices, zoning for fast‐food businesses) is crucial to justify and inform systems‐based method selection and decisions. Determining whether the problem is simple, complicated, or complex facilitates a deeper understanding of the problem and prepares researchers for the degree of uncertainty that increases with the complexity of the problem (e.g., using the Cynefin framework [3]). Complex systems and problems typically exhibit many characteristics (Table 1). Because of this, they regularly call for participatory methods involving subject matter experts, change agents, and policymakers who can articulate, interpret and work effectively with complexity, including industry, government, community members, and non‐governmental agencies.

Upon clarifying that the problem is complex, understanding *why* it persists and the intended outcomes and benefits of using a system science approach will guide method selection. Problem articulation is a core step across all methods to confirm purpose, scope and modelling requirements, including boundary conditions and variable selection. This could be completed using a ‘problem framing matrix’ as found in community‐based system dynamics [57, 58], which contains four distinct categories, including: (i) learning problems (i.e., individuals/communities cannot learn or adapt to the changing situation or experience); (ii) coordination problems (i.e., conflict among actors, e.g., no shared consensus); (iii) transformation problems (i.e., problems persists because of the underlying system structure); and (iv) analysis problems (i.e., policies are objectively wrong given system constraints in the real world). Capturing the type of problem will inform method decisions, such as stakeholder input, system components, and the modelling process, but can shift throughout the project by learning more about the problem/system.

Third, once researchers confirm that an approach matches their intuition, problem complexity and goals, they should consult key literature for that method, using tools like the intuitive‐to‐formal continuum to gauge how easy it is to begin [53]. Methods and tools that have been identified as being relatively intuitive include rich pictures (i.e., drawing the system), theory of change models (i.e., draw/write the connections between an intervention and outcomes) and constructing a causal loop diagram (i.e., up to the formal hypothesis of system dynamics) [53]. Once the appropriate approach is selected, transparent reporting, from system conceptualisation and analysis through to formal model development, is essential to advance the application of systems science methods in nutrition and dietetics. Systems thinking reflects a worldview and skillset developed through repeated exposure and practice but demands regular and reflective engagement to promote fluency across the field. Embedding systems thinking into tertiary nutrition and dietetic programs and professional development opportunities is necessary to advance the discipline's response to contemporary real‐world challenges.

As confidence in using systems‐based approaches in research grows, these insights need to be integrated into education and professional development to enhance how the workforce is professionalised. Beyond upskilling at the individual level, integrating systems thinking into nutrition and dietetics education and professional culture more broadly is pivotal to equip the workforce with the mindset and ability to navigate complex issues [59]. Systems‐based practice receives little attention in health professional competencies, with dietetic competencies currently reflecting an awareness of broader social and environmental influences, but limited articulation of systems approaches and collaborating across disciplines to challenge broader systems [60]. Whilst there is a need to update curricula, there are also opportunities to promote early engagement through the guiding principles described above, and further enhancing the learning through embedding systems thinking into existing professional frameworks (e.g., the nutrition care process), emphasising experiential learning and progressively building fluency over time [25].

## 4 Conclusion

Traditional scientific paradigms have made important contributions to nutrition and dietetics research, particularly in supporting individual‐level interventions; however, there is a need to complement these paradigms with systems‐based approaches that better account for the complexity of real‐world nutrition and dietetic challenges. Creating meaningful, widespread change in nutrition outcomes requires us to leverage the benefits of such approaches to facilitate a more comprehensive understanding and ability to address complex problems. Greater engagement with and integration of systems science is needed to build on the noted existing exemplars and strengthen the capacity of the discipline for complex problem‐solving. Agent‐based modelling, system dynamics, and network analysis represent three prominent methods to conceptualise, explore and analyse complex problems. Early engagement and confidence using systems science can be achieved through (i) awareness of the plurality of approaches; (ii) aligning methods with the problem space; and (iii) allowing systems fluency to develop through research adoption and integration into education. Strengthening the nutrition and dietetics research workforce's capacity to embrace complexity is pivotal to driving transformative, scalable improvements in population nutrition and health.

## Author Contributions

Study conceptualisation: all (K.T., A.H., M.R., A.K., L.B.). Writing – original: K.T. & A.H. Writing – editing: all (K.T., A.H., M.R., A.K., L.B.).

## AI Transparency Statement

AI assistance (Microsoft Co‐pilot) was used only for language refinement and editing of original author‐generated content.

## Conflicts of Interest

The authors declare no conflicts of interest.

## Data Availability

Data sharing not applicable to this article as no datasets were generated or analyzed during the current study.
